# Thermal–Hydrodynamic Behavior and Design of a Microchannel Pin-Fin Hybrid Heat Sink

**DOI:** 10.3390/mi13122136

**Published:** 2022-12-02

**Authors:** Xiaonan Guan, Zhihui Xie, Gang Nan, Kun Xi, Zhuoqun Lu, Yanlin Ge

**Affiliations:** 1 College of Power Engineering, Naval University of Engineering, Wuhan 430033, China; 2 Institute of Thermal Science and Power Engineering, Wuhan 430205, China; 3 School of Mechanical & Electrical Engineering, Wuhan Institute of Technology, Wuhan 430205, China

**Keywords:** thermal–hydrodynamic coupling, 3D stacked chip, hybrid heat sink, microchannel, pin-fins

## Abstract

A three-dimensional convective heat transfer model of a microchannel pin-fin hybrid heat sink was established. Considering the non-uniform heat generation of 3D stacked chips, the splitting distance of pin-fins was optimized by minimizing the maximum heat sink temperature under different heat fluxes in the hotspot, the Reynolds numbers at the entrance of the microchannel, and the proportions of the pin-fin volume. The average pressure drop and the performance evaluation criteria were considered to be the performance indexes to analyze the influence of each parameter on the flow performance and comprehensive performance, respectively. The results showed that the maximum temperature of the hybrid heat sink attained a minimum value with an increase in the splitting distance. The average pressure drop in the center passage of the microchannel first increased and then decreased. Furthermore, the optimal value could not be simultaneously obtained with the maximum temperature. Therefore, it should be comprehensively considered in the optimization design. The heat flux in the hotspot was positively correlated with the maximum heat sink temperature. However, it had no effect on the flow pressure drop. When the Reynolds number and the pin-fin diameter increased, the maximum heat sink temperature decreased and the average pressure drop of the microchannel increased. The comprehensive performance of the hybrid heat sink was not good at small Reynolds numbers, but it significantly improved as the Reynolds number gradually increased. Choosing a bigger pin-fin diameter and the corresponding optimal value of the splitting distance in a given Reynolds number would further improve the comprehensive performance of a hybrid heat sink.

## 1. Introduction

Electronic devices are developing toward miniaturization, being lightweight, and having a high integration, promoted by the developments in science and technology. A high-level integration leads to a high-power consumption density per unit volume. The performance of a chip is substantially affected if the large amount of heat generated by the integrated circuit cannot be removed in time. Furthermore, thermal failure may occur in severe cases. The proposed microchannel provides a solution to this problem [1].

The heat generation of chips in practical engineering applications is occasionally non-uniform. This leads to the formation of a “hotspot” in the local areas of chips. Certain studies have shown that the heat flux in a hotspot area can be over eight times the average heat flux in the background area [2]. This easily leads to a great non-uniform temperature distribution in an electronic device, reducing the service life of the system. Accordingly, it is of high practical significance to optimize the design of a hybrid heat sink that integrates multiple areas with different cooling capacities (hereinafter referred to as a “hybrid heat sink”); e.g., the combination of a microchannel heat sink in the background area and a pin-fin heat sink in the hot area. In addition, an in-chip cooling structure that is embedded in a semiconductor can reduce the parasitic thermal resistance and improve the overall heat dissipation effect [3,4]. Therefore, an interlayer microchannel pin-fin hybrid heat sink has a broad application prospect for meeting the challenges from high-power microchip-level thermal management problems.

Scholars worldwide have carried out a substantial amount of research on microchip-level cooling technology [5,6,7,8,9,10,11,12,13]. Ansari et al. [14] proposed a hybrid micro-heat sink for cooling microprocessors with non-uniform heat generation. The temperature inhomogeneity and maximum temperature were chosen as the performance indexes as well as the thermal resistance and pump power. Comparative analyses were performed between these with those of a conventional microchannel heat sink. Wang et al. [15] employed a large eddy simulation to investigate the heat transfer performance and flow characteristics of a rectangular channel with three-row miniature square column vortex generators when the Reynolds number varied from 3745 to 11,235. Ling et al. [16] designed a novel interlaced microchannel. Two sidewalls of the microchannel functioned as the main heat transfer surface. The thermal and hydraulic performance of the interlaced and parallel microchannels was numerically investigated using a full-sized conjugate heat transfer model and then compared with the experimental results. The optimized geometric dimensions of the interlaced microchannel, including the depth, width, and spacing, were obtained. Feng et al. [17] investigated the effects of several fluid flow parameters and double-sided heating power on the performance of a heat sink with embedded microfins by experimental and numerical simulation methods, taking the temperature uniformity as the optimization objective. Cheng et al. [18] investigated the hydrodynamic and thermal characteristics of a slot array in a microchannel based on a three-dimensional numerical simulation, and a comparison with a seamless array was conducted. Ding et al. [19] analyzed the temperature distribution at the bottom of a processor and the flow field distribution inside a microchannel for a 3D-IC sandwich microchannel structure. Xie et al. [20] studied the effects of an inclined angle and arrangement method on the hydraulic and thermal performance of microchannel heat sinks. Chen et al. [21] presented a novel cross-rib microchannel heat sink to induce a fluid to self-rotate and then studied the effects of the aspect ratio of the microchannel. Omri et al. [22] presented a microchannel heat exchanger equipped with triangular fins and applied a CNT–water nanofluid as a coolant. They found that the triangular fins in the heat exchanger could significantly improve the thermal performance of the microchannel heat exchanger and the geometrical parameters of the triangular fins had various effects on the comprehensive performance of the microchannel heat exchanger.

Based on the literature [14,17,18], this study applied split pin-fins to a hybrid heat sink; considered the thermal–hydrodynamic coupling effect of, and the non-uniform heat generation by, the chip; optimized the splitting distance of the pin-fins; and analyzed the influence of the heat flux on the hotspot, the Reynolds number at the entrance of the microchannel, and the volume ratio of the pin-fins on the thermal–hydrodynamic performance of the chip. The results may provide theoretical guidance for the design of a high-power microchip-level thermal management scheme.

## 2. Models and Solutions

### 2.1. Geometrical Model

**Figure 1:**
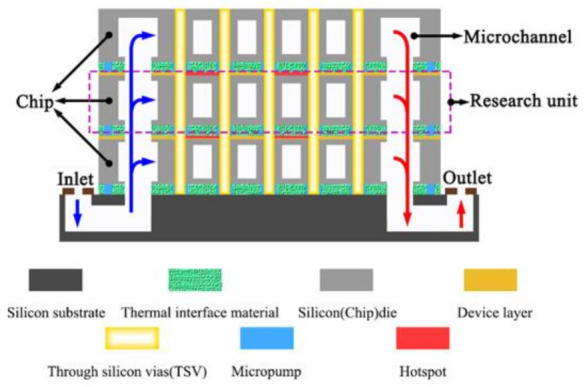
Schematic diagram of 3D stacked chips with microchannel hybrid heat sink [11].

**Figure 2:**
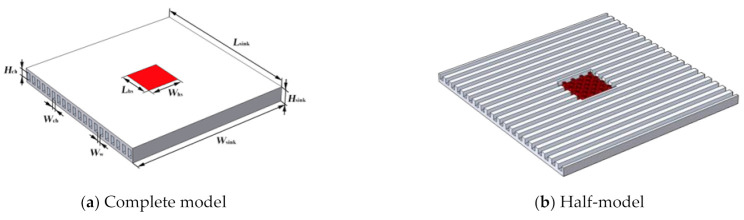
Geometric model of microchannel pin-fin hybrid heat sink [9]. (**a**) Complete model of heat sink. (**b**) Half-model of heat sink.

Figure 1 shows a 3D stacked chip with a microchannel interlayer cooling system. As shown in the figure, each chip layer had an independent microchannel interlayer cooling the heat sink. The microchannels and pin-fins arrays were embedded to enhance the heat dissipation effect. Each chip was connected by thermal interface material (TIM) and a micropump. The TSV integrated in the microchannel sidewall and pin-fin arrays enabled an electrical and communication interconnection between the different chips. The silicon substrate was the base that supported the entire 3D stacked chip and acted as a fluid inlet and outlet. The liquid coolant was pumped through a micropump into the fluid inlet. It then flowed through the hybrid heat sink and finally into the fluid outlet. As shown in Figure 1 [11], each chip layer had a device layer that generated heat and transferred it to the embedded hybrid heat sink. Thus, an individual hybrid heat sink (see Figure 2a [9]) was heated on both sides. To simplify the model and reduce the computational cost, an individual hybrid heat sink was selected for an optimization design and the boundary conditions of the double-sided heat flow were specified. In addition, the heat flux on both sides of the hybrid heat sink was non-uniformly distributed to account for the non-uniform heat generation of the chip. The heated area was divided into the hot area and the background area. The heat flux in the hot area was significantly higher than that in the background area.

The heat sink was integrated with 20 microchannels and 25 pin-fins, which were mainly divided into two areas. The part around the heat sink was the background area of the low heat flux cooled by the microchannels. The red part in the center was the hot area of the high heat flux cooled by the pin-fin array. The values of the geometric parameters of the model are presented in Table 1.

**Figure 3:**
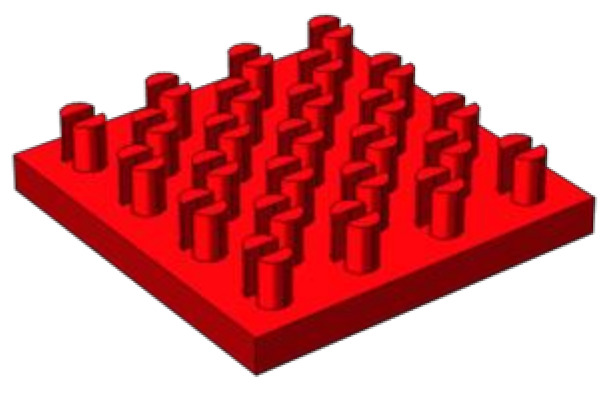
Geometric model of hotspot area.

**Figure 4:**
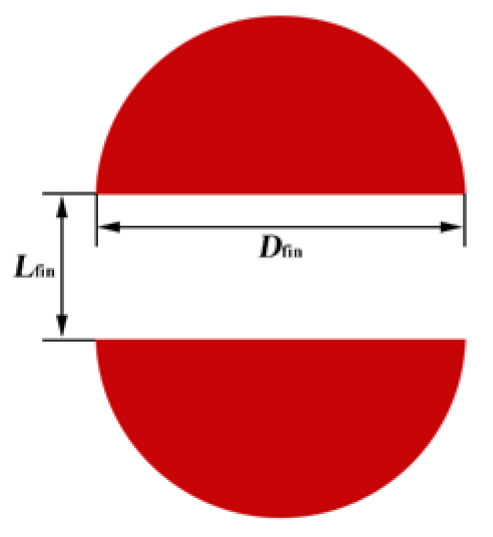
Schematic diagram of split pin-fin cross-section.

A heat sink was used for the interlayer cooling of the chip. The upper and lower surfaces were heated equally and the remaining outer surface was insulated. In this example, the heat flux in the background area was *q*_bg_ = 50 W·cm^−2^, the heat flux in the hot area was *q*_hs_ = 400–600 W·cm^−2^, and the Reynolds number at the entrance of the microchannel was *Re* = 100–300. The proportion of the pin-fin volume was controlled by the pin-fin diameter, which was *D*_fin_ = 100–260 μm in this example. As the structure and boundary conditions of the model were horizontally symmetrical, only half of the model was developed and calculated for simplification (see Figure 2b). Figure 3 and Figure 4 show a hotspot model diagram and a cross-sectional diagram of the split pin-fins used in this study, respectively.

### 2.2. Physical Model

The material of the microchannel pin-fin hybrid heat sink was silicon (constant pressure specific heat capacity *c*_p,s_ = 700 J·kg^−1^·k^−1^, density *ρ*_s_ = 2329 kg·m^−3^, and thermal conductivity *k*_s_ = 130 W·m^−1^·k^−1^). The cooling medium was deionized water with constant physical parameters. The cooling medium flowed in from one end of the heat sink and flowed out from the other end after a convective heat transfer with the high-temperature wall and high-temperature fins. The Reynolds number at the inlet was set to $Re=U_{\mathrm{in}}D_{h}/v$ = 100~300, in which *U*_in_ (m·s^−1^) was the inlet velocity of the deionized water, $D_{h}=2W_{\mathrm{ch}}\cdot H_{\mathrm{ch}}/\left(W_{\mathrm{ch}}+H_{\mathrm{ch}}\right)$ (m) was the hydraulic diameter of the microchannel, and *ν* (m^2^·s^−1^) was the kinematic viscosity of the deionized water. The pressure boundary condition at the exit was *p*_out_ = 1 atm pressure. The upper and lower surfaces of the rectangular heat sink were heated equally and the remaining outer surface was insulated. In this example, the heat flux from the background area was *q*_bg_ = 50 W·cm^−2^ and the heat flux from the hotspot area was *q*_hs_ = 400~600 W·cm^−2^. At the fluid–solid interface, there was no slip in the fluid velocity (i.e., *U* = 0) and the temperature was seen as continuous (i.e., *T*_s_= *T*_w_ and $q”=\lambda_{s}\partial T_{s}/\partial n=\lambda_{f}\partial T_{f}/\partial n$). The other external surfaces were adiabatic surfaces (i.e., ∂T/∂n=0).

The heat flux in the background area and hotspot area was uniform:

(1) $$q^{\prime \prime }=-k_{s}{\left(\nabla T\right)}_{s}$$

The continuity equation, momentum equation, and energy equation of the fluid laminar flow were:

(2) $$\nabla \cdot \left(\rho U\right)=0$$

(3) $$\rho \left(U\cdot \nabla \right)U=\nabla \cdot \left[-pI+\mu \left(\nabla U+{\left(\nabla U\right)}^{T}\right)-\frac{2}{3}\mu \left(\nabla \cdot U\right)I\right]+F$$

(4) $$\rho c_{p}U\cdot \nabla^{T}+\nabla \cdot q=Q$$

where *ρ* (kg·m^−3^) is the density, **U** (m·s^−1^) is the velocity vector, *p* (Pa) is the pressure, **I** is the identity matrix, **F**(N) is the volume force vector, **q** (W·m^−2^) is the heat flux vector, *Q* (W·m^−2^) is the heat source term (including the viscous dissipation and pressure work), and *T* denotes the matrix transpose operation.

### 2.3. Numerical Methods and Grid Tests

**Figure 5:**
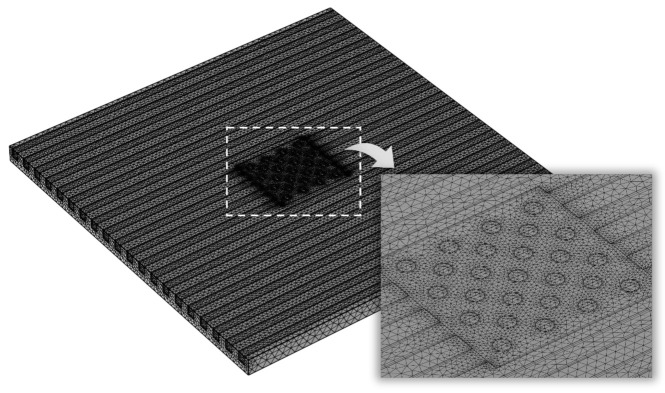
Schematic diagram of meshes generated in the microchannel pin-fin hybrid heat sink.

In this study, the finite element calculation software COMSOL Multiphysics was used to solve the thermal–hydrodynamic-coupled model; the flow viscous dissipation was considered whereas the gravitational force and radiation were neglected. The unstructured tetrahedral mesh was divided into fluid and solid areas; the denser boundary layer mesh was divided into areas near the fluid–solid interfaces. The independence of the grid was tested to ensure the calculation accuracy. Considering *q*_hs_ = 500 W·cm^−2^, *Re* = 200, *D*_fin_ = 180 μm, and *L*_fin_ = 100 μm as an example, the number of grids under the three partitioning strategies of coarsening, conventional, and refinement were 276,205, 873,442, and 2,965,967, respectively. The corresponding *T*_max_ was 365.93 K, 368.96 K, and 371.31 K, and the relative errors were 0.828% and 0.637%, respectively. A second mesh division strategy was adopted in this study to consider the calculation accuracy and efficiency. Figure 5 shows the schematic diagram of the meshes generated in the microchannel pin-fin hybrid heat sink, where the hotspot area was partially enlarged. The common default convergence criterion was used for the continuity, momentum, and energy equations.

**Figure 6:**
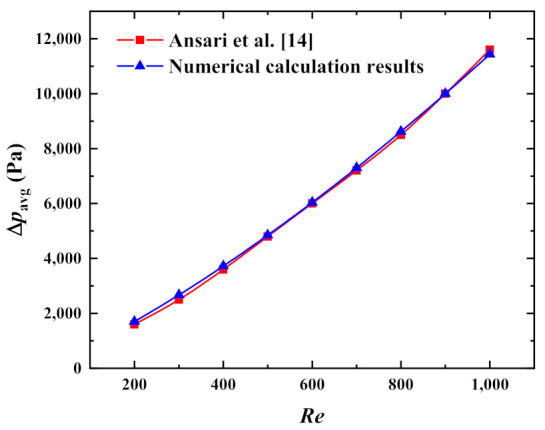
Verification of model validity in the maximum pressure drop versus *Re*.

To further verify the accuracy of the algorithm in this study, the modeling method in this study was used to establish the microchannel pin-fin hybrid heat sink and heat dissipation model developed in [14] and its calculation was carried out. When the other conditions were fixed, the parameter relationship between the maximum pressure drop of the cooling channels and the Reynolds number was obtained, as shown in Figure 6. As is evident from the numerical comparison, the deviations between the maximum pressure drop of the cooling channel in [14] and the simulation model in this study were negligible at 9 Reynolds numbers.

## 3. Results and Discussion

### 3.1. Thermal Behavior and Optimization

(1) With different heat fluxes at the hotspot

**Figure 7:**
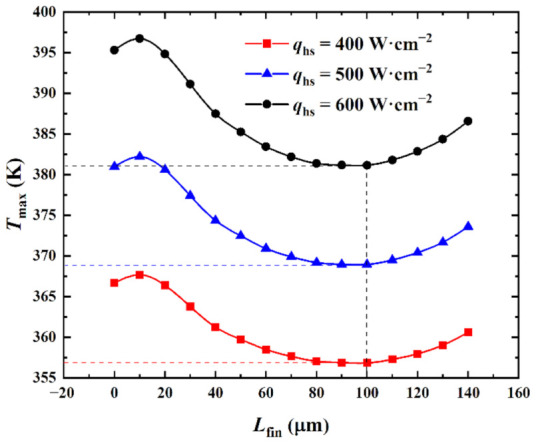
Effect of q_hs_ on *T*_max_ versus *L*_fin_.

Figure 7 shows the influence of the heat flux *q*_hs_ in the hotspot on the relationship between the maximum temperature *T*_max_ and the splitting distance *L*_fin_ when the inlet Reynolds number was *Re* = 200 and the pin-fin diameter was *D*_fin_ = 180 μm.

Figure 7 shows that when *q*_hs_ was specified and as *L*_fin_ increased, *T*_max_ first marginally increased, then substantially decreased, and finally increased again. The fluid flow rate in the interlayer of the split pin-fins was highly marginal when *L*_fin_ was marginal. This was due to the influence of the boundary layer. At this time, the main heat transfer mode was heat conduction and the convective heat transfer made a limited contribution to the heat dissipation effect. Simultaneously, the vertical streamline length of the pin-fins increased, the flow stagnation area of the back flow side of the pin-fins increased, and the heat dissipation performance decreased. Thus, *T*_max_ increased with the increase in *L*_fin_. As *L*_fin_ continued to increase, the flow rate of the fluid flowing through the interlayer of the pin-fins gradually increased and the main heat transfer mode became a convective heat transfer. The heat transfer performance of the pin-fins was enhanced and *T*_max_ decreased accordingly. When *L*_fin_ > 100 μm, the fluid flow on both sides of the pin-fins and the heat dissipation performance on both sides of the surface continued to decrease with the increase in *L*_fin_. This caused *T*_max_ to gradually increase.

A comparison of the relationship curves between *T*_max_ and *L*_fin_ at different *q*_hs_ revealed that the curves for *q*_hs_ = 400 W•cm^−2^, 500 W•cm^−2^, and 600 W•cm^−2^ had a similar variation trend. Here, the curves first marginally increased, then substantially decreased, and finally increased again. *T*_max_ minima were obtained at (*L*_fin_)_opt_ = 100 μm for the three curves under different *q*_hs_ conditions. This indicated that *q*_hs_ had no effect on the thermal behavior of the element body. The extreme values of *T*_max_ for the three curves increased with the increase in *q*_hs_ (i.e., 356.85 K, 368.96 K, and 381.16 K, respectively) with increments of 12.11 K and 12.20 K, respectively. It was observed that when *q*_hs_ increased, *T*_max_ proportionally increased; these were positively correlated. This was because the increase in *q*_hs_ increased the heat output of the chip and the heat dissipation load of the heat sink. If the generated heat was not emitted in time, *T*_max_ accordingly increased until a thermal balance was attained. (*T*_max_)_min_ under different *q*_hs_ was reduced by 9.84 K, 12.04 K, and 14.16 K, respectively, compared with *T*_max_ = 366.69 K, 381.00 K, and 395.32 K under *L*_fin_ = 0 μm (reduction of 2.68%, 3.16%, and 3.58%, respectively). That is, the larger the value of *q*_hs_, the more significant the effect of *L*_fin_ on *T*_max_. This implied that the higher the heat flux of the 3D stacked chip, the more significant the optimization effect of varying the needle rib splitting distance on the maximum temperature.

(2) With different Reynolds numbers at the microchannel entrance

**Figure 8:**
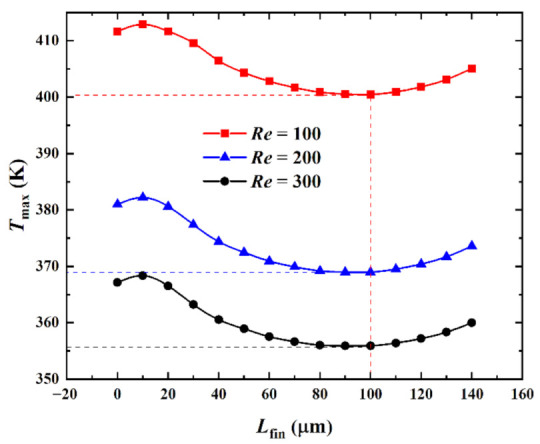
Effect of *Re* on *T*_max_ versus *L*_fin_.

Figure 8 shows the influence of the inlet Reynolds number (*Re*) on the relationship between the maximum temperature *T*_max_ and the splitting distance *L*_fin_ when the heat flux in the hotspot was *q*_hs_ = 500 W·cm^−2^ and the pin-fin diameter was *D*_fin_ = 180 μm.

Figure 8 shows that when the *Re* was specified and as *L*_fin_ increased, *T*_max_ marginally increased, then decreased, and finally increased again. A comparison between the relationship curves of *T*_max_ and *L*_fin_ for different *Re* revealed that the curves for *Re* = 100, 200, and 300 had a similar trend. When *Re* differed, the three curves achieved a *T*_max_ minima at (*L*_fin_)_opt_ = 100 μm with extreme values of 400.46 K, 368.96 K, and 355.93 K, respectively, and incremental values of −31.50 K and −13.03 K, respectively. The overall heat transfer coefficient *h*_avg_ corresponding with these *T*_max_ minima were 30,290 W·m^−2^·K^−1^, 35,077 W·m^−2^·K^−1^, and 38,432 W·m^−2^·K^−1^, respectively. It was observed that for the same model, the fluid flow rate increased and the convective heat transfer effect was enhanced when *Re* increased. This, in turn, improves the heat dissipation performance of the heat sink and decreased *T*_max_. The larger the value of *Re*, the smaller the effect of varying *Re* on *T*_max_. That is, an appropriate increase in *Re* when it was small could reduce *T*_max_ more effectively. (*T*_max_)_min_ under different *Re* was reduced by 11.19 K, 12.04 K, and 11.19 K compared with *T*_max_ = 411.65 K, 381.00 K, and 367.12 K, respectively, for *L*_fin_ = 0 μm (reduction of 2.72%, 3.16%, and 3.05%, respectively). Therefore, when *T*_max_ was used as a performance metric, variations in *T*_max_ were less affected by *Re* as *L*_fin_ varied.

(3) With different volume ratios (pin-fin diameters)

**Figure 9:**
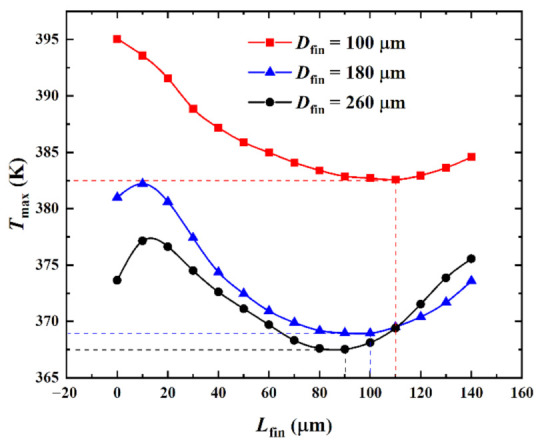
Effect of *D*_fin_ on *T*_max_ versus *L*_fin_.

**Figure 10:**
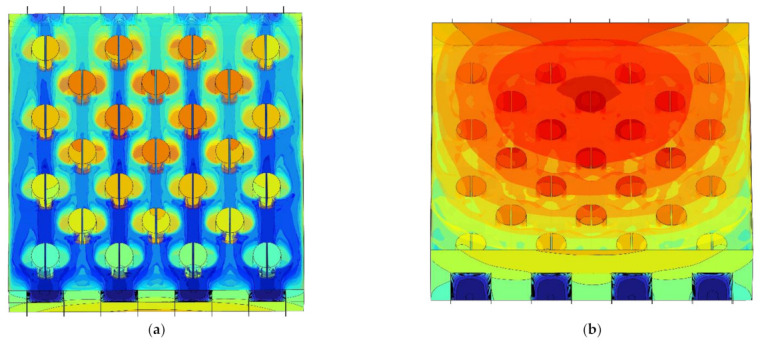

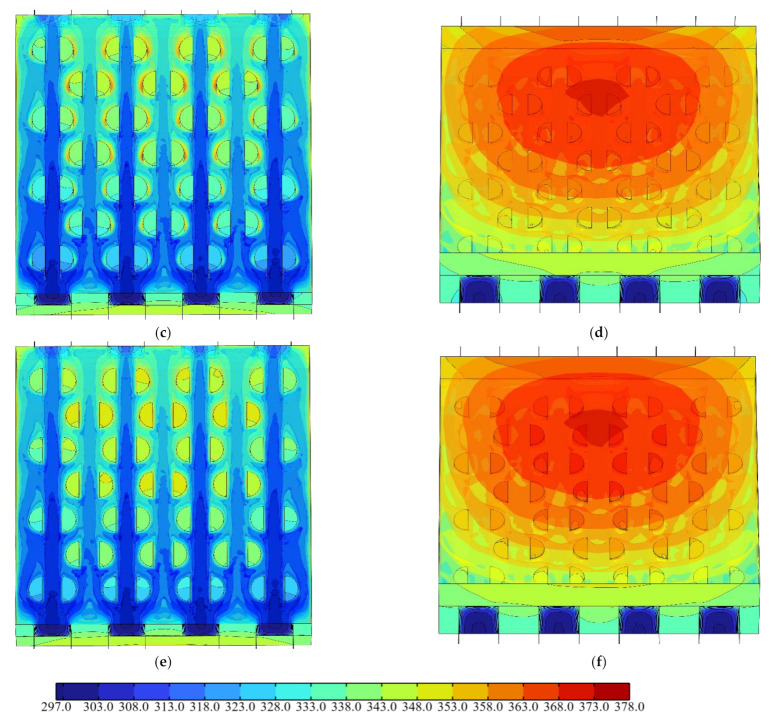
Temperature color maps of hotspot area of the hybrid heat sink with different *L*_fin_ (unit K). (**a**) *L*_fin_ = 10 μm (top view). (**b**) *L*_fin_ = 10 μm (bottom view). (**c**) *L*_fin_ = 100 μm (top view). (**d**) *L*_fin_ = 100 μm (bottom view). (**e**) *L*_fin_ = 140 μm (top view). (**f**) *L*_fin_ = 140 μm (bottom view).

Figure 9 shows the influence of the pin-fin diameter *D*_fin_ on the relationship between the maximum temperature *T*_max_ and the splitting distance *L*_fin_ when the heat flux in the hotspot was *q*_hs_ = 500 W·cm^−2^ and the inlet Reynolds number was *Re* = 200. Corresponding with Figure 9, Figure 10 shows the temperature color maps of the hotspot area of the hybrid heat sink when *D*_fin_ = 180 μm and *L*_fin_ = 10, 100, and 140 μm, respectively.

As can be observed from Figure 9, when *D*_fin_ = 180 μm and 260 μm and as *L*_fin_ increased, *T*_max_ marginally increased, then decreased, and finally increased. When *D*_fin_ = 100 μm and as *L*_fin_ increased, *T*_max_ first decreased and then increased. When *D*_fin_ = 180 μm and 260 μm, *T*_max_ passed through three stages as *L*_fin_ increased. The first stage was where *L*_fin_ was small. Here, the main heat transfer mode in the interlayer of the split pin-fins was heat conduction owing to the influence of the small fluid flow rate in the boundary layer. *T*_max_ marginally increased with the increase in *L*_fin_. When *D*_fin_ = 180 μm, the *T*_max_ maximum in the first stage was 381.96 K and the corresponding overall heat transfer coefficient *h*_avg_ was 33,612 W·m^−2^·K^−1^. In the second stage, *L*_fin_ continued to increase, the flow-around effect increased, the disturbance of the fluid flow increased, the convective heat transfer effect increased and became the dominant heat transfer mode, and *T*_max_ accordingly decreased. When *D*_fin_ = 180 μm, the *T*_max_ minimum in the second stage was 368.60 K and the corresponding overall heat transfer coefficient *h*_avg_ was 35,067 W·m^−2^·K^−1^. The third stage was when *L*_fin_ increased to a certain value. With the increase in *L*_fin_, the fluid mainly flowed through the interlayer of the pin-fins and the convective heat transfer effect on the semicircular surface of the pin-fins weakened. This resulted in an increase in *T*_max_. When *D*_fin_ = 180 μm and *L*_fin_ = 140 μm, *T*_max_ was 373.23 K and the corresponding overall heat transfer coefficient *h*_avg_ was 34,527 W·m^−2^·K^−1^. When *D*_fin_ = 100 μm, the obtained data did not reflect the first stage of *T*_max_ because the numerical simulation calculation step (10 μm) was large. These showed a decreasing trend followed by an increasing trend with the increase in *L*_fin_.

When *D*_fin_ was 100 μm, 180 μm, and 260 μm, *T*_max_ attained a minimum value when (*L*_fin_)_opt_ = 110 μm, 100 μm, and 90 μm, respectively. The extreme values were 382.57 K, 368.96 K, and 368.13 K, respectively, and the increments were −13.61 K and −0.83 K, respectively. *T*_max_ was reduced by 12.48 K, 12.04 K, and 5.52 K compared with *T*_max_ = 395.05 K, 381.00 K, and 373.65 K, respectively, for *L*_fin_ = 0 μm (reduction of 3.15%, 3.16%, and 1.48%, respectively). It was observed that with the increase in *D*_fin_, the surface area of the pin-fin heat transfer and the heat transfer increased. The smaller the value of (*L*_fin_)_opt_, the less optimal the *T*_max_ that was obtained by varying *L*_fin_. Furthermore, the smaller the value of *D*_fin_, the more significant the effect of varying *D*_fin_ and *L*_fin_ was on *T*_max_. This indicated that *D*_fin_ and *L*_fin_ had optimal matching issues when *T*_max_ was considered to be the performance index. Therefore, in practical engineering applications, the pin-fin diameter can be determined by comprehensively considering the cost, process, and other factors. (*L*_fin_)_opt_ can then be determined according to the relationship curve between *L*_fin_ and *T*_max_ so that the heat sink *T*_max_ is minimized and the performance is optimal.

### 3.2. Hydrodynamic Behavior Analysis

(1) Influence of the heat flux at the hotspot

**Figure 11:**
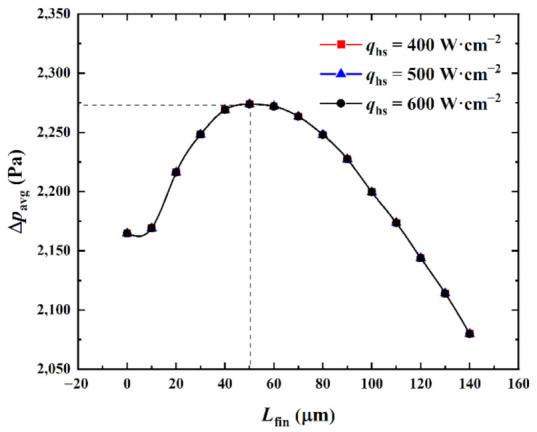
Effect of *q*_hs_ on ∆*p*_avg_ versus *L*_fin_.

Figure 11 shows the influence of the heat flux *q*_hs_ at the hotspot on the relationship between the average pressure drop ∆*p*_avg_ of the center passage and the splitting distance *L*_fin_ when the inlet Reynolds number was *Re* = 200 and the pin-fin diameter *D*_fin_ = 180 μm.

As can be observed from Figure 11, when *q*_hs_ was given and as *L*_fin_ increased, ∆*p*_avg_ marginally increased, then substantially increased, and finally continuously decreased. When *L*_fin_ was small, the fluid flow in the interlayer of the split pin-fins was gradual and the disturbance was weak owing to the influence of the boundary layer. However, the increase in the thickness of the split pin-fins in the vertical streamline direction obstructed the fluid flow. This marginally increased the flow resistance and ∆*p*_avg_ increased with it. When *L*_fin_ > 10 μm and continued to increase, the fluid flow in the split pin-fin interlayer gradually increased, so that part of the fluid flowed through the split pin-fin interlayer, the disturbance was enhanced, the flow resistance increased, and ∆*p*_avg_ greatly increased. When *L*_fin_ > 50 μm and with the increase in *L*_fin_, the fluid flow on both sides of the pin-fins gradually decreased, the fluid mainly flowed through the interlayer of the split pin-fins, the disturbance weakened, and ∆*p*_avg_ continued to decrease.

A comparison of the relationship curves between ∆*p*_avg_ and *L*_fin_ for different *q*_hs_ revealed that the curves for *q*_hs_ = 400 W·cm^−2^, 500 W·cm^−2^, and 600 W·cm^−2^ essentially overlapped. Moreover, ∆*p*_avg_ attained a maximum when (*L*_fin_)_opt_ = 50 μm. A minimum value of 2079.9 Pa was obtained at (*L*_fin_)_opt_ = 140 μm. This was 84.8 Pa lower than ∆*p*_avg_ = 2164.7 Pa for the cylindrical pin-fins at *L*_fin_ = 0 μm (a reduction of 3.92%). It was observed that the variation in *q*_hs_ essentially had no effect on ∆*p*_avg_. This was because the effect of the variation in *q*_hs_ only altered the temperature field. However, the microchannel retained a single-phase incompressible laminar flow (*Re* = 200; the fluid was liquid and the physical properties did not vary with the temperature). Thus, the variation in the temperature field at a certain range had no effect on the fluid flow and the flow field and ∆*p*_avg_ were not affected by *q*_hs_. In addition, from the perspective of the fluid flow, increasing the pin-fin splitting distance to the maximum extent within the range permitted by the manufacturing process could be considered. This would reduce the pressure drop in the flow passage and the pump power consumption.

(2) Influence of the Reynolds number at the microchannel entrance

**Figure 12:**
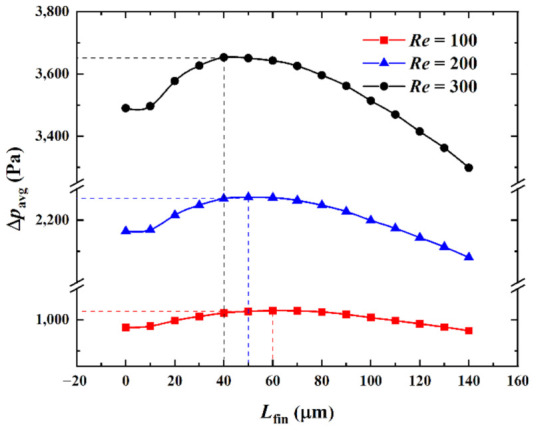
Effect of *Re* on ∆*p*_avg_ versus *L*_fin_.

Figure 12 shows the influence of the Reynolds number (*Re*) at the entrance of the microchannel on the relationship between the average pressure drop ∆*p*_avg_ in the center passage and the splitting distance *L*_fin_ when the heat flux in the hotspot was *q*_hs_ = 500 W·cm^−2^ and the pin-fin diameter was *D*_fin_ = 180 μm.

As can be observed from Figure 12, when *Re* was specified and as *L*_fin_ increased, ∆*p*_avg_ marginally increased, then substantially increased, and finally continuously decreased. A comparison of the relationship curves of ∆*p*_avg_ and *L*_fin_ for different *Re* revealed that the curves for *Re* = 100, 200, and 300 had a similar variation trend. ∆*p*_avg_ attained the maximum when (*L*_fin_)_opt_ = 60 μm, 50 μm, and 40 μm and the respective minimums were 964.7 Pa, 2079.9 Pa, and 3298.0 Pa when (*L*_fin_)_opt_ = 140 μm, respectively. The increments were 1115.2 Pa and 1218.1 Pa, which were 9.8 Pa, 84.8 Pa, and 191.8 Pa lower than ∆*p*_avg_ = 974.5 Pa, 2164.7 Pa, and 3489.8 Pa when *L*_fin_ = 0 μm. The reduction percentages were 1.00%, 3.92%, and 5.50%, respectively. It was observed that the flow velocity and flow resistance increased as *Re* increased and ∆*p*_avg_ required by the flow accordingly increased. The higher the value of *Re*, the higher the impact of ∆*p*_avg_ on the *Re* variations. That is, the effect of reducing the heat sink ∆*p*_avg_ by reducing *Re* was better when *Re* was small. In addition, when *Re* was large, the fluid flow rate was large, the disturbance was strong, the fluid was substantially affected by the variation in the pin-fin geometric parameters, *L*_fin_ had a more significant effect on ∆*p*_avg_, and the optimization space was larger. Therefore, in engineering applications, the higher the value of *Re*, the higher the significance of the optimal design related to the pin-fin splitting distance.

(3) Influence of the volume ratio (pin-fin diameter)

**Figure 13:**
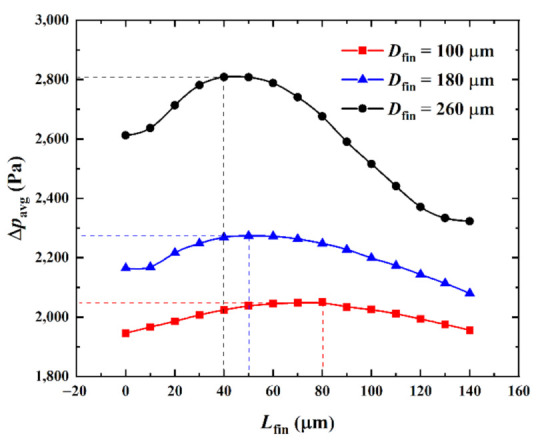
Effect of *D*_fin_ on ∆*p*_avg_ versus *L*_fin_.

**Figure 14:**
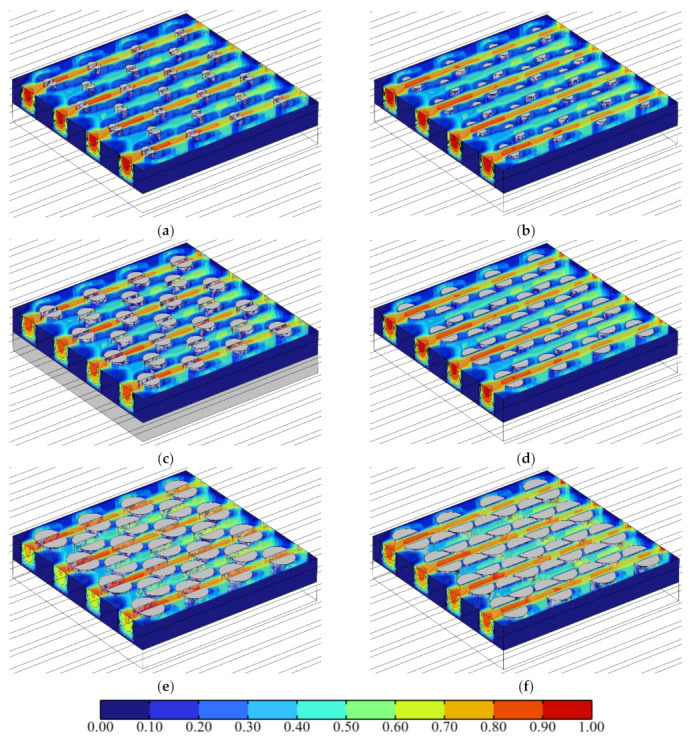
Velocity color maps of hotspot area of the hybrid heat sink with different *L*_fin_ (unit m·s^−1^). (**a**) *D*_fin_ = 100 μm, *L*_fin_ = 40 μm. (**b**) *D*_fin_ = 100 μm, *L*_fin_ = 140 μm. (**c**) *D*_fin_ = 180 μm, *L*_fin_ = 50 μm. (**d**) *D*_fin_ = 180 μm, *L*_fin_ = 140 μm. (**e**) *D*_fin_ = 260 μm, L_fin_ = 80 μm. (**f**) *D*_fin_ = 260 μm, *L*_fin_ = 140 μm.

Figure 13 shows the influence of the pin-fin diameter *D*_fin_ on the relationship between the average pressure drop ∆*p*_avg_ in the center passage and the splitting distance *L*_fin_ when the heat flux in the hotspot was *q*_hs_ = 500 W·cm^−2^ and the inlet Reynolds number was *Re* = 200. Corresponding with Figure 13, Figure 14 shows the velocity color maps of the hotspot area of the hybrid heat sink when ∆*p*_avg_ reached the maxima and minima at *D*_fin_ = 100, 180, and 260 μm, respectively.

As can be observed from Figure 13, as *L*_fin_ increased from 0 μm to 140 μm, the fluid flow in the microchannel underwent a process wherein it mainly flowed through both sides of the pin-fins to the interlayer of the split pin-fins. The fluid disturbance first increased and then weakened. ∆*p*_avg_ varied with it; increasing first and then continuously decreasing. A comparison between the relationship curves of ∆*p*_avg_ and *L*_fin_ with different *D*_fin_ revealed that for *D*_fin_ = 100 μm, 180 μm, and 260 μm, ∆*p*_avg_ attained the maximum value when *L*_fin_ = 80 μm, 50 μm, and 40 μm, respectively. Meanwhile, the minimum values were obtained when *L*_fin_ = 0 μm, 140 μm, and 140 μm (namely, 1946.1 Pa, 2079.9 Pa, and 2322.7 Pa, respectively, with increments of 133.8 Pa and 242.8 Pa, respectively). Compared with ∆*p*_avg_ = 1946.1 Pa, 2164.7 Pa, and 2612.4 Pa for *L*_fin_ = 0 μm, it was reduced by 0 Pa, 84.8 Pa, and 289.7 Pa, respectively (i.e., by 0%, 3.92%, and 11.09%, respectively). It was observed that the larger the *D*_fin_, the smaller the minimum value of ∆*p*_avg_. Furthermore, the larger the decrease with the increase in *D*_fin_, the larger the variation in the influence of *L*_fin_. That is, the larger the *D*_fin_, the higher its influence on ∆*p*_avg_. Compared with the cylindrical pin-fin hybrid heat sink when *L*_fin_ =0 μm and when ∆*p*_avg_ was taken as the performance index, the larger the *D*_fin_, the better the optimal performance that could be achieved by tweaking *L*_fin_ for the heat sink. In addition, when *D*_fin_ = 100 μm, the minimum value of ∆*p*_avg_ was *L*_fin_ = 0 μm. The cylindrical pin-fins then formed the optimal configuration.

### 3.3. Comprehensive Performance of the Microchannel Pin-Fin Hybrid Heat Sink

From the above thermal and hydrodynamic behavior analysis of the microchannel pin-fin hybrid heat sink, the influences of the flow and structure parameters on the maximum temperature *T*_max_ and the overall heat transfer coefficient *h*_avg_ of the hybrid heat sink were exactly contrary to the average pressure drop ∆*p*_avg_ of the center passage of the hybrid heat sink. This indicated that it was necessary to further confirm if the comprehensive performance of the microchannel pin-fin hybrid heat sink was better than the conventional microchannel heat sink.

The performance evaluation criteria $PEC=(Nu_{a}\;/\;Nu_{0})\;/\;\left({\left(f_{a}\;/\;f_{0}\right)}^{1/3}\right)$ is a well-known and widely used indicator to describe the comprehensive performance of heat sinks [23]. *Nu* is the overall Nusselt number, *f* is the apparent flow friction coefficient, the subscript “0” indicates the conventional microchannel heat sink, and the subscript “a” indicates the microchannel pin-fin hybrid heat sink.

When the heat flux in the hotspot *q*_hs_ = 500 W·cm^−2^ and the Reynolds number *Re* = 100~300, the apparent flow friction coefficients *f*_0_ of the conventional microchannel heat sink with the exact dimensions as Table 1 were 0.1652, 0.0871, and 0.0591, respectively; the overall Nusselt numbers *Nu*_0_ of the conventional microchannel heat sink were 13.958, 15.674, and 16.842, respectively. The above parameters were used as the benchmark to compare the comprehensive performance of the microchannel pin-fin hybrid heat sink.

Considering the effect of the Reynolds number (*Re*) and the splitting distance *L*_fin_ on the comprehensive performance of the microchannel pin-fin hybrid heat sink (*D*_fin_ = 180 μm), *PEC* = 0.8595 when (*L*_fin_)_opt_ = 100 μm at *Re* = 100, *PEC* = 1.2248 when (*L*_fin_)_opt_ = 100 μm at *Re* = 200, and *PEC* = 1.5131 when (*L*_fin_)_opt_ = 100 μm at *Re* = 300, respectively. This indicated that the comprehensive performance of the microchannel pin-fin hybrid heat sink was not good at small Reynolds numbers, but could be significantly improved as the Reynolds number gradually increased.

Considering the effect of the pin-fin diameter *D*_fin_ and the splitting distance *L*_fin_ on the comprehensive performance of the microchannel pin-fin hybrid heat sink (*q*_hs_ = 500 W·cm^−2^; *Re* = 200), *PEC* = 1.1607 when (*L*_fin_)_opt_ = 110 μm at *D*_fin_ = 100 μm, *PEC* = 1.2217 when (*L*_fin_)_opt_ = 100 μm at *D*_fin_ = 180 μm, and *PEC* = 1.2312 when (*L*_fin_)_opt_ = 100 μm at *D*_fin_ = 180 μm, respectively. This indicated that, at a given Reynolds number, the thermal performance and the comprehensive performance of the microchannel pin-fin hybrid heat sink could be further improved with a bigger pin-fin diameter *D*_fin_ and corresponding optimal value of the splitting distance (*L*_fin_)_opt_.

## 4. Conclusions

In this study, split pin-fins were applied to a hybrid heat sink and the thermal–hydrodynamic behavior and optimal design of a split pin-fin array at the hotspot was obtained. The influence of the heat flux at the hotspot, the Reynolds number at the entrance of the microchannel, and volume proportion of the pin-fins were analyzed.

The results showed that the maximum temperature of the hybrid heat sink had a minimum value with the increase in the splitting distance of the pin-fins. The average pressure drop in the center passage of the microchannel first increased and then decreased. An optimal value could not be simultaneously obtained with the maximum temperature. Therefore, it should be comprehensively considered in the optimization design. The heat flux in the hotspot was positively correlated with the maximum heat sink temperature. However, it had no effect on the flow pressure drop. The effects of the Reynolds number at the entrance of the microchannel and the volume proportion of the pin-fins were similar. That is, when the Reynolds number and the volume proportion increased, the maximum heat sink temperature decreased whereas the average pressure drop of the microchannel increased and the comprehensive performance of the microchannel pin-fin hybrid heat sink was improved. The larger the value of the Reynolds number and the volume proportion, the smaller the effect.

Based on the concept of a thermal–hydrodynamic coupling optimization design, this study provides a new method and example for the optimization design of a hybrid heat sink with a microchannel and pin-fins. Considering the different processes and structural forms of microchannels and pin-fins, further research to carry out a multi-objective optimization design, including non-uniform heat production and its dynamic characteristics, is worthwhile.

## Figures and Tables

**Table 1:** Dimensions of model.

*L*_sink_	*W*_sink_	*H*_sink_	*L*_hs_	*W*_hs_	*W*_ch_	*H*_ch_	*W*_w_
10,000 μm	10,000 μm	900 μm	2000 μm	2000 μm	250 μm	500 μm	250 μm

## Data Availability

Data will be made available on request.
